# Copper-catalysed alkylation of heterocyclic acceptors with organometallic reagents

**DOI:** 10.3762/bjoc.16.90

**Published:** 2020-05-14

**Authors:** Yafei Guo, Syuzanna R Harutyunyan

**Affiliations:** 1 Stratingh Institute for Chemistry, University of Groningen, Nijenborgh 4, 9747 AG, Groningen, The Netherlands

**Keywords:** conjugate addition, copper catalysis, heterocyclic Michael acceptor, organometallics

## Abstract

Copper-catalysed asymmetric C–C bond-forming reactions using organometallic reagents have developed into a powerful tool for the synthesis of complex molecules with single or multiple stereogenic centres over the past decades. Among the various acceptors employed in such reactions, those with a heterocyclic core are of particular importance because of the frequent occurrence of heterocyclic scaffolds in the structures of chiral natural products and bioactive molecules. Hence, this review focuses on the progress made over the past 20 years for heterocyclic acceptors.

## Introduction

The copper-catalysed asymmetric addition of organometallic reagents to various acceptors is a useful strategy for C–C bond-forming reactions [1–4]. These important transformations have been thoroughly developed in the last few decades and were widely used in the synthesis of chiral natural products and bioactive molecules [5–8]. The majority of these molecules has a crucial commonality, namely the presence of heterocyclic units containing nitrogen, oxygen, sulphur, or other heteroatoms. These units are also often responsible for the key bioactivities that such molecules exhibit [9–11]. This has motivated the development of various strategies that target the synthesis of chiral heterocyclic motives [12–14]. Among these, methodologies based on the copper-catalysed asymmetric addition of organometallics are especially valuable because of i) the compatibility between copper catalysts and heteroatoms present in the starting materials that often show inhibitory effects in combination with other metal-based catalysts, and ii) the availability and cost-efficiency of copper(I) salts and most organometallics.

This review aims to provide an overview on the copper-based catalytic systems that enable the direct application of heterocyclic acceptors in highly enantioselective C–C bond-forming reactions with organometallics. The work highlighted in this minireview is divided into two sections, based on the position where the bond is formed. The first part focuses on acceptors in which the reacting unsaturated double bond is embedded into the heterocyclic ring, while the second part deals with acceptors in which the reacting unsaturated double bond is located outside of the heterocyclic unit (e.g., alkenyl-substituted heterocycles). The organometallics discussed in this minireview include organoaluminium, organozinc, organozirconium, organolithium, and Grignard reagents.

## Review

### Copper-catalysed C–C bond-forming reactions at the heterocycle

The direct synthesis of chiral heterocyclic molecules from pyridine, quinolone, or indole derivatives is advantageous due to the abundance of such building blocks. Unfortunately, establishing catalytic enantioselective methods for the synthesis of these compounds resulting in high yield and enantioselectivity has proven challenging. As a result, significant effort has been invested into copper-catalysed asymmetric conjugate addition reactions using organometallics.

**Scheme 1:**
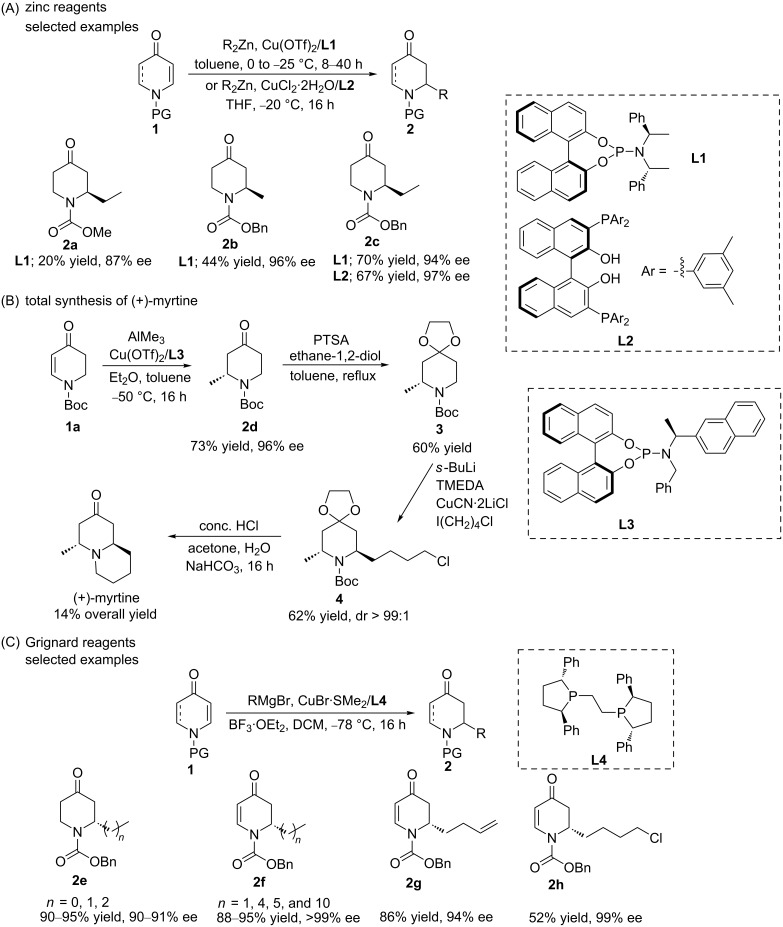
Copper-catalysed ACA of organometallics to piperidones. A) addition of organozinc reagents; B) addition of organoaluminium reagent in the total synthesis of (+)-myrtine; C) addition of Grignard reagents.

In 2005, Feringa and co-workers reported on the copper-catalysed asymmetric conjugate addition (ACA) of dialkylzinc reagents to *N*-substituted 2,3-dehydro-4-piperidones **1** in order to access useful chiral piperidine derivatives (Scheme 1A) [15]. They found the catalytic system based on the chiral phosphoramidite **L1** and a copper salt to be the most efficient one to achieve an enantioselectivity of up to 96% ee*.*

Interestingly, piperidones with different carbamate protecting groups (Me, Et, Ph, tosyl, and Bn, respectively) were tolerated, and a high enantioselectivity could also be obtained with several other dialkylzinc reagents (e.g., iPr_2_Zn and *n-*Bu_2_Zn, respectively). Later, T. Shibata and K. Endo prepared the same product (**2c**) with a higher enantioselectivity (97% ee) by using the multinuclear phosphorus ligand catalyst **L2** [16]. Organoaluminium reagents are also commonly used organometallics in copper-catalysed ACA reactions. For example, in the work of Feringa and co-workers, the methylation reaction using Me_2_Zn resulted in a low yield of 44% due to the difficult purification of the crude product [15]. However, the same authors showed later that the copper-catalysed ACA of Me_3_Al to Boc-protected 4-piperidone can be used as a key step in the total synthesis of the natural product (+)-myrtine with 14% overall yield (Scheme 1B) [17]. For this application, the highest yield (73%) and enantioselectivity (96% ee) were obtained using the chiral ligand **L3** and a copper salt as the catalyst.

Despite the fact that examples of high yield and enantioselectivity have been reported for conjugate additions of both organoaluminium and organozinc reagents, these reagents also present major drawbacks, namely their commercial availability and atom efficiency, given that only one alkyl group is transferred from the organometallic reagent to the Michael acceptor. In contrast, Grignard reagents are very favourable organometallics in terms of both their availability and atom efficiency. On the other hand, Grignard reagents are significantly more reactive than organoaluminium and organozinc reagents, rendering the catalytic control of both the regio- and enantioselectivity in addition reactions challenging. Nevertheless, Harutyunyan and co-workers introduced the first general catalytic methodology to access a wide variety of chiral piperidones in 2019, using Grignard reagents (Scheme 1C) [18]. Therein, a new catalytic system based on the ligand **L4**/Cu complex promoted the addition of Grignard reagents to *N*-Cbz-pyridone and *N*-Cbz-2,3-dihydropyridone Michael acceptors with high enantioselectivity and yield. It is worth mentioning that in copper-catalysed additions of Grignard reagents to *N*-Cbz-pyridone, the use of a Lewis acid (BF_3_·OEt_2_) together with the copper catalyst is essential for achieving a high yield as well as a high regio- and enantioselectivity (up to 99% ee).

**Scheme 2:**
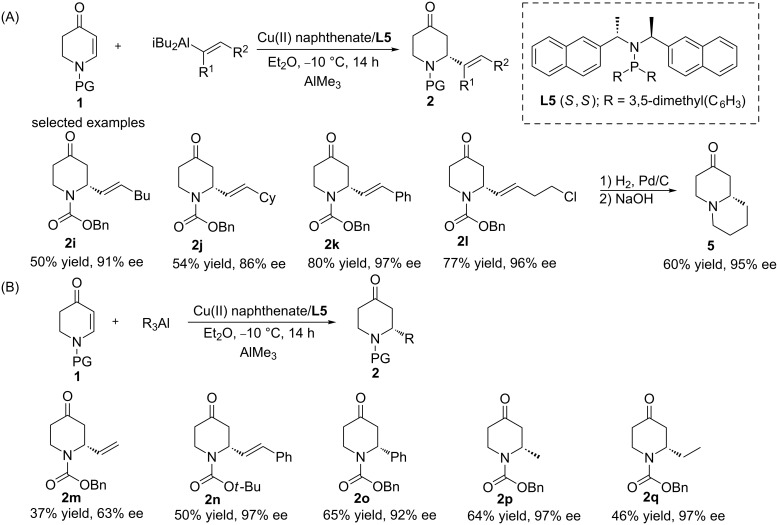
Copper-catalysed ACA of alkenylalanes to N-substituted-2,3-dehydro-4-piperidones.

Although organoaluminium, organozinc, and Grignard reagents were all successfully applied in the ACA of 2,3-dehydro-4-piperidones, an introduction of the vinyl group was not successful until 2012, when Alexakis and co-workers disclosed that vinylalanes could be used in the copper-catalysed ACA to *N*-substituted-2,3-dehydro-4-piperidones [19]. Optimisation studies revealed that the combination of the ligand **L5** and Cu(II) naphthenate constituted the most efficient catalytic system, allowing the synthesis of the corresponding products with good yield and enantiomeric purity (up to 83% yield and 97% ee). Furthermore, a large variety of vinylalanes was investigated, and the product **2l** was further derivatised into a chiral bicyclic structure (**5**, Scheme 2A). In addition, simple vinyl aluminium reagents and commercial alkylaluminium reagents were examined in this methodology, providing the corresponding products with a moderate yield but high enantioselectivity (Scheme 2B).

**Scheme 3:**
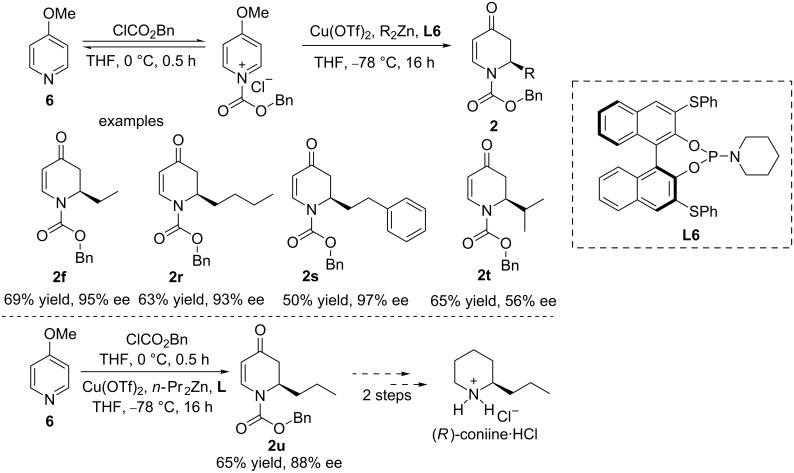
Copper-catalysed asymmetric addition of dialkylzinc reagents to *N*-acyl-4-methoxypyridinium salts formed in situ.

In 2009, Feringa and co-workers presented the first highly enantioselective 1,2-addition of dialkylzinc reagents to an *N*-acyl-4-methoxypyridinium salt using a copper/phosphoramidite catalytic system (Scheme 3) [20–21]. The authors highlighted that the *N*-acylpyridinium salts were unstable species and that their instability affected the regioselectivity of the dearomatisation process upon the nucleophilic addition of the organozinc reagents. To solve this problem, the intermediate of the *N*-acyl-4-methoxypyridinium salt must be formed in situ and added slowly to the solution of the Cu(OTf)_2_/**L6** complex and dialkylzinc reagent at −78 °C. Several dialkylzinc reagents were found to be effective as nucleophiles in this reaction, in most cases providing the products with a high enantioselectivity and a moderate yield. An exception was found with diisopropylzinc, for which only 56% ee could be obtained. The methodology was also successfully applied to the total synthesis of the natural alkaloid (*R*)-coniine.

**Scheme 4:**
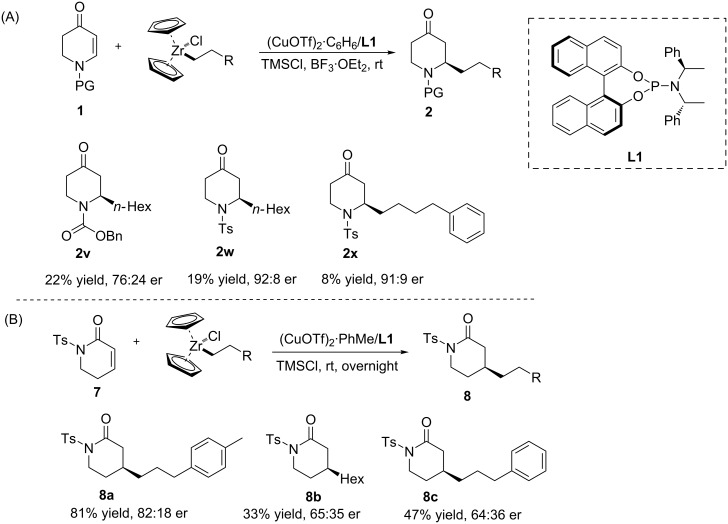
Copper-catalysed ACA of organozirconium reagents to N-substituted 2,3-dehydro-4-piperidones and lactams.

Organozirconium compounds are another class of organometallic reagents that have been used widely in the synthesis of complex molecules. Recently, Fletcher and co-workers demonstrated the applicability of a hydrozirconation in the ACA reaction to non-heterocyclic conjugated substrates [22–26], while the Šebesta group was the first to report the copper-catalysed addition of organozirconium reagents to *N*-substituted 2,3-dehydro-4-piperidones (Scheme 4A) [27]. In the latter work, the organozirconium reagents were generated first in situ by the hydrozirconation of alkenes. Subsequently, the **L1**/Cu catalytic system was used to test different organozirconium reagents. The results showed that with *N*-substituted 2,3-dehydro-4-piperidones, several products could be obtained with an enantiomeric ratio of up to 92:8, but with yields not exceeding 22%. Interestingly, this methodology could also be applied to the lactams **7**, leading to the corresponding products with up to 81% yield and an enantiomeric ratio of up to 82:18 (Scheme 4B).

**Scheme 5:**
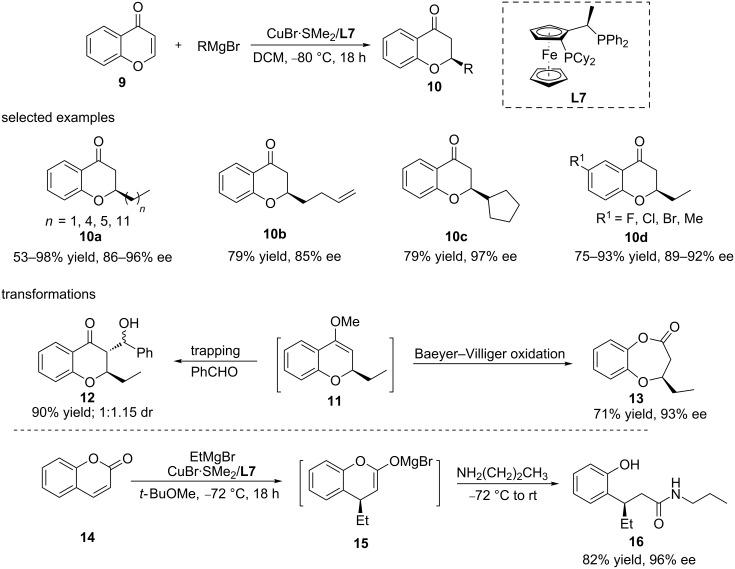
Copper-catalysed ACA of Grignard reagents to chromones and coumarins and further derivatisation of the corresponding products.

Oxygen-containing heterocyclic compounds are ubiquitous in natural products and medicines, with many of them being chiral. Copper-catalysed ACA reactions of organometallics have also been employed to synthesize such chiral oxygen-containing heterocyclic compounds. Feringa’s group reported the highly regio- and enantioselective copper-catalysed direct conjugate addition of Grignard reagents to chromones and coumarins (Scheme 5) [28–29]. A variety of Grignard reagents, including linear and secondary alkylmagnesium reagents and various chromones and coumarins, were tolerated by the catalytic system, providing the products with a high yield and enantioselectivity. It was also demonstrated that the addition products could be used for further transformations in order to access various derivatives, such as **12** and **13**, derived from the trapping and Baeyer–Villiger oxidation of **11**, respectively, or compound **16**, obtained via the ring opening reaction of **15** with an amine (Scheme 5). Taking the enolate intermediate derived from the addition of EtMgBr to coumarin as an example, it was shown that upon the treatment with an amine, this enolate produced the final chiral amide product with a good yield (82%) and ee (96%).

**Scheme 6:**
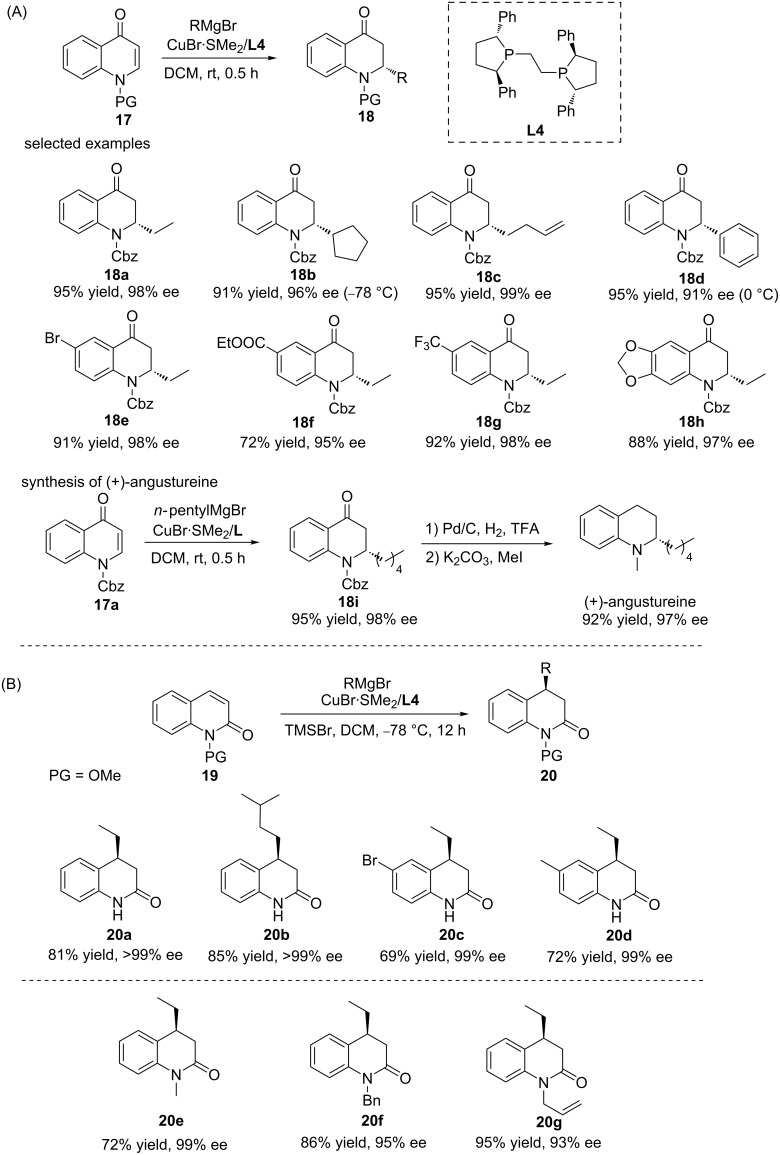
Copper-catalysed ACA of Grignard reagents to N-protected quinolones.

While the methodology for the ACAs of Grignard reagents to chromones and coumarins has been established successfully, quinolones remained challenging substrates for such transformations until very recently. It was not until 2019 that this problem was solved, when the Harutyunyan group employed a catalytic system based on **L4**/Cu, which efficiently catalysed the ACA of Grignard reagents to N-protected quinolones **17** at room temperature (Scheme 6) [18]. Initially, the methodology was developed for additions to *N*-Cbz-4-quinolone-based substrates, and the catalytic system was demonstrated to facilitate the addition of a wide variety of reagents, including linear, α-, β-, and γ-substituted, as well as aryl Grignard reagents. The subsequent broadening of the quinolone scope revealed that substrates bearing Me, Br, CF_3_, ether, amide, and ester substituents, respectively, were also tolerated successfully. In addition, the catalytic system was applied to the synthesis of the natural product (+)-angustureine with an excellent outcome (92% yield, 97% ee) (Scheme 6A). When the method was applied to the ACA of Grignard reagents to N-substituted 2-quinolones, their lower reactivity led to a lower conversion. Performing the reaction in the presence of TMSBr resolved this and allowed the reaction to proceed for various Grignard reagents and substrates with an excellent enantioselectivity (Scheme 6B).

**Scheme 7:**
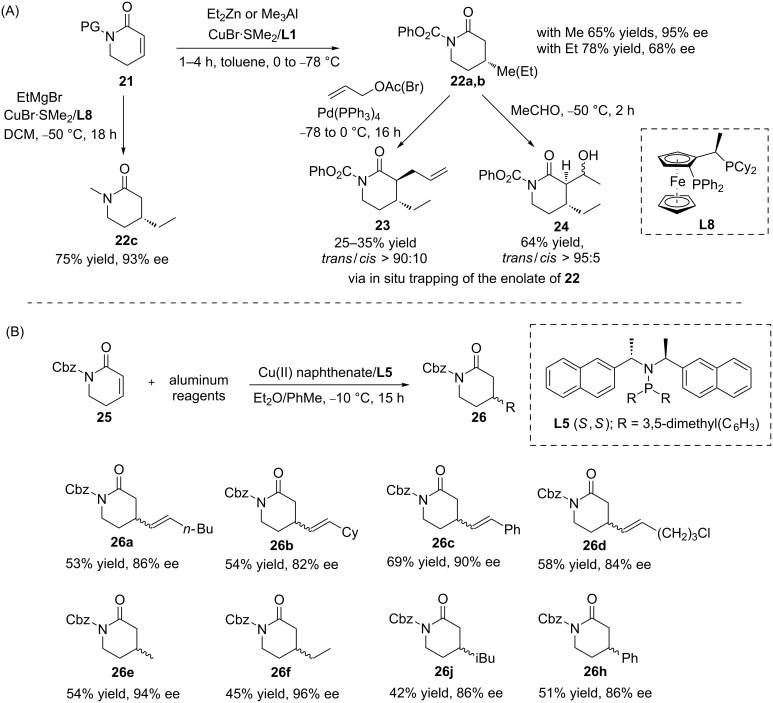
Copper-catalysed ACAs of organometallics to conjugated unsaturated lactams.

The copper-catalysed ACA of organometallics has also been applied to lactams, which are useful building blocks for synthetic chemistry. In 2004, Pineschi and co-workers successfully introduced the methodology of copper-catalysed ACAs of organoaluminium and organozinc reagents to lactams (Scheme 7A) [30]. They found that with a phenylcarbamate protecting group on the nitrogen atom, the addition of Et_2_Zn and Me_3_Al could be promoted by the **L1**/Cu catalytic system, leading to the corresponding alkylated products with 95% and 68% enantioselectivity, respectively. Furthermore, the intermediate formed upon this ACA could be trapped with acetaldehyde and allyl bromide or allyl acetate to form valuable compounds with high ee and dr values. Later, Harutyunyan’s research group showed that also non-activated lactams with alkyl-protected groups could undergo ACA reactions with EtMgBr, with 93% ee (**22c**, Scheme 7A) [31]. The research group of Alexakis was able to push this chemistry further when they developed a new methodology that allowed to access the chiral lactams **26** with moderate yield and high enantioselectivity (up to 96% ee). Therein, the copper(II) naphthenate/**L5**-catalysed ACA of alkenylaluminium and alkylaluminium reagents to the β-substituted unsaturated conjugated lactams **25** was utilized (Scheme 7B) [32].

**Scheme 8:**
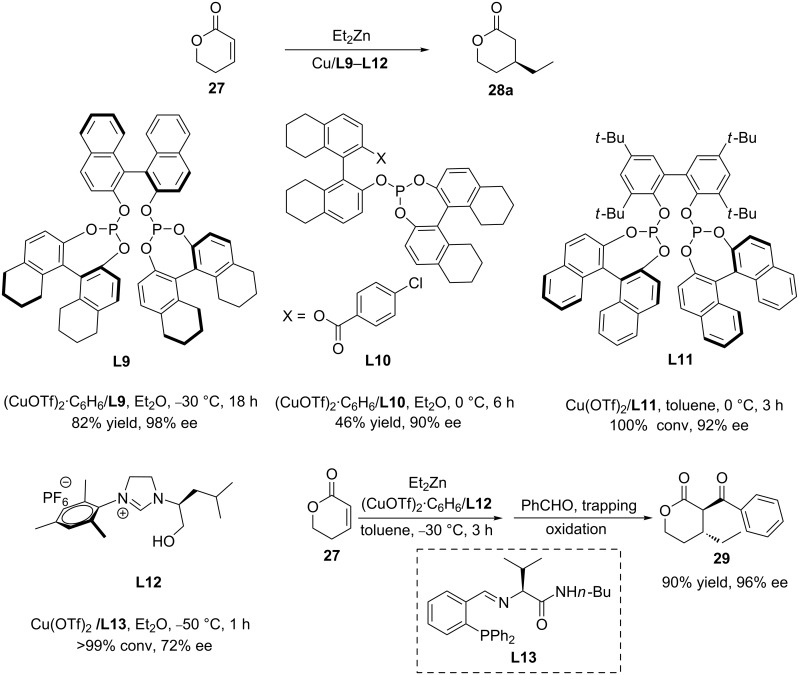
Copper-catalysed ACA of Et_2_Zn to 5,6-dihydro-2-pyranone.

Chiral lactones are yet another interesting class of heterocyclic substrates that have attracted great attention because of their usefulness in both organic synthesis and medicinal chemistry. The copper-catalysed ACA of organozinc reagents to 5,6-dihydro-2-pyranone is one of the best methods to obtain chiral lactones. During the past two decades, the research groups of Chan, Hoveyda (who applied an approach depending on the trapping by an aldehyde), Mauduit, and Wang reported a variety of methods employing the chiral ligands **L9**–**L13** that, in combination with copper, efficiently catalysed the ACA of diethylzinc to 5,6-dihydro-2-pyranone (Scheme 8), providing access to chiral β-substituted lactones with high enantioselectivity and conversion [33–37].

**Scheme 9:**
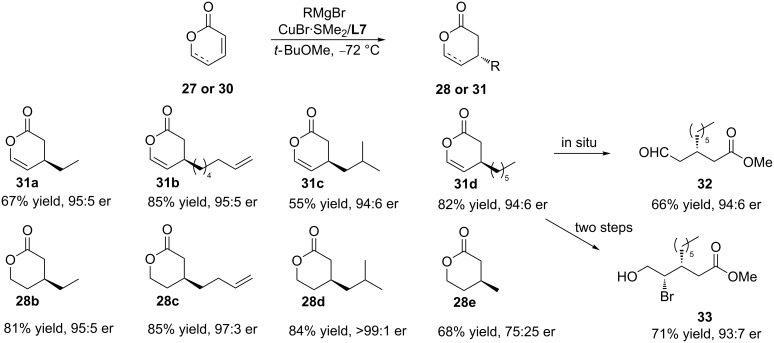
Copper-catalysed ACA of Grignard reagents to pyranone and 5,6-dihydro-2-pyranone.

Although the copper-catalysed ACA of Et_2_Zn to 5,6-dihydro-2-pyranone has been reported, the reactivity and commercial availability of the former renders the ACA of Grignard reagents a more attractive methodology. Feringa and co-workers were the first to report copper-catalysed ACAs of alkyl Grignard reagents to pyranones and 5,6-dihydro-2-pyranone (Scheme 9) [38]. In the presence of the Cu/**L7** catalytic system, several alkyl Grignard reagents underwent ACAs to form the chiral lactones with high enantioselectivity. Importantly, the authors showed how the conjugate addition products could be further derivatised to lead to versatile chiral building blocks, such as a β-alkyl-substituted aldehyde (66% yield, 94:6 er) or a β-bromo-γ-alkyl-substituted alcohol (71% yield, 93:7 er).

**Scheme 10:**
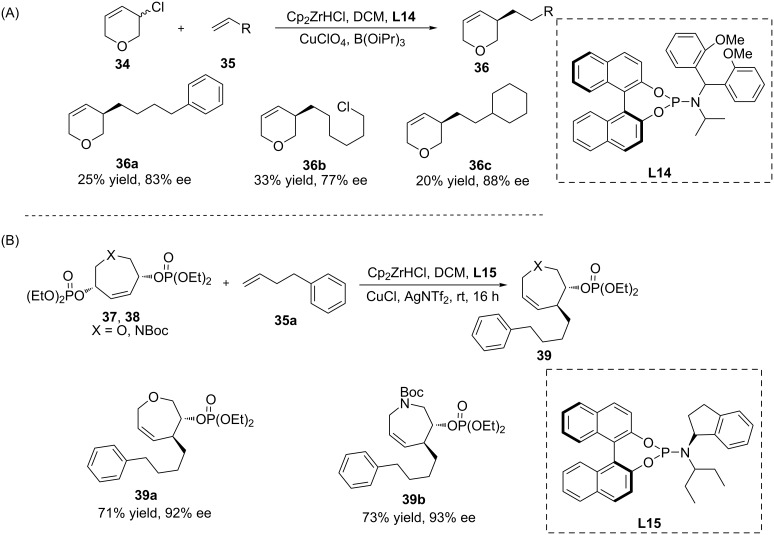
Copper-catalysed AAA of an organozirconium reagent to heterocyclic acceptors.

The asymmetric allylic alkylation (AAA) is a very useful method that allows the enantioselective formation of C–C bonds, and copper-catalysed AAAs using Grignard, organolithium, organoaluminium, and organozirconium reagents have been reported. In 2015, Fletcher and co-workers presented the copper-catalysed AAA of racemic 3,6-dihydro-2*H*-pyrans using alkylzirconocenes in the presence of the Cu/**L14** catalytic system (Scheme 10A) [39]. Several alkylzirconocenes were examined, resulting in the respective products with 45–93% ee and 20–33% yield. The same group also described the copper-catalysed desymmetrisation of heterocyclic meso compounds via the AAA reaction, once again using alkylzirconocenes as nucleophiles (Scheme 10B). In this reaction, two seven-membered heterocyclic bisphosphates (O- and N-containing, respectively,) underwent Cu/**L15**-catalysed AAAs and provided the corresponding chiral products with good yield and high enantioselectivity (92–93% ee) [40].

**Scheme 11:**
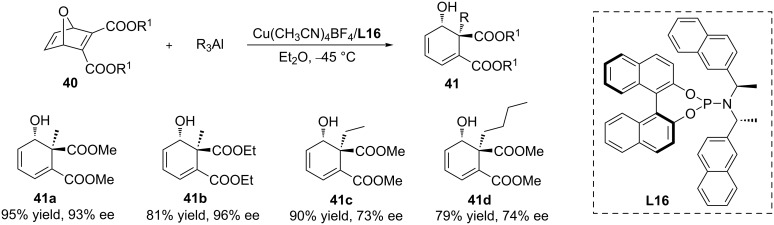
Copper-catalysed ring opening of an oxygen-bridged substrate with trialkylaluminium reagents.

Ring opening reactions where carbon–carbon bonds are formed upon the addition of organometallics to heterocyclic acceptors, resulting in products that are not heterocyclic, provide an alternative strategy to generate important building blocks with two stereocentres, starting from heterocyclic substrates. The copper-catalysed ring opening of oxygen-bridged heterocyclic acceptors with trialkylaluminium reagents was explored by the group of Alexakis in 2009 (Scheme 11) [41]. Various chiral phosphoramidite ligands, in combination with a copper salt, were found to be efficient catalysts for this transformation, with the best results obtained with the ligand **L16**.

**Scheme 12:**
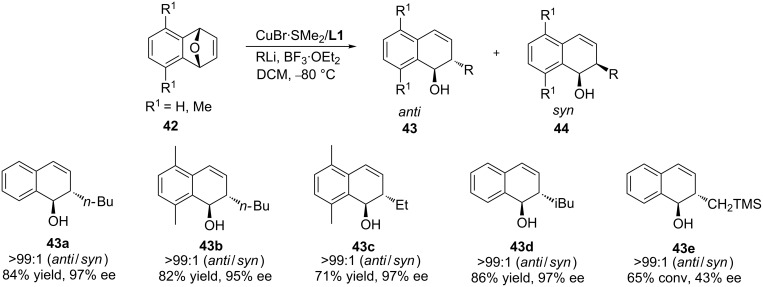
Copper-catalysed ring opening of oxabicyclic substrates with organolithium reagents (selected examples).

Feringa and co-workers elaborated the copper-catalysed ring opening reaction of oxabicyclic alkene substrates using organolithium reagents, finding excellent *anti* selectivities and enantioselectivity (Scheme 12) [42]. During the optimisation studies, they discovered that when the Lewis acid BF_3_·OEt_2_ was employed in combination with the Cu/**L1** catalyst system, the *anti* diastereoisomer could be obtained with 97% enantioselectivity. In addition, this methodology tolerated *n-*BuLi, iBuLi, *n-*HexLi, and EtLi, providing a full conversion and high *anti* selectivity and enantioselectivity in all cases.

**Scheme 13:**
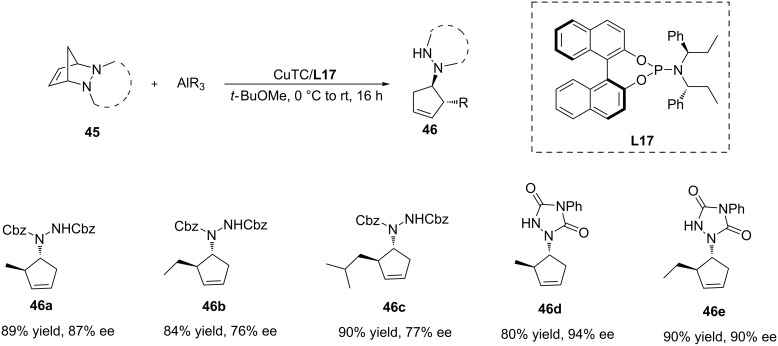
Copper-catalysed ring opening of polycyclic meso hydrazines.

Alexakis and co-workers exploited the copper-catalysed asymmetric ring opening of polycyclic meso hydrazines with organoaluminium reagents (Scheme 13) [43]. This reaction followed a classical allylic substitution pathway. Interestingly, the organoaluminium reagents in this reaction did not only act as alkyl donors but could also activate the leaving group. After testing several kinds of phosphoramidite ligands with copper salts, the catalyst system **L17**/CuTc was selected for further studies. The solvent was found to play a crucial role in this reaction, with MTBE as the solvent of choice. Various organoaluminium reagents and protecting groups were examined, providing the products with good yield (up to 90%) and ee (up to 95%).

### Copper-catalysed conjugate addition reactions to alkenyl-substituted heterocycles

**Scheme 14:**
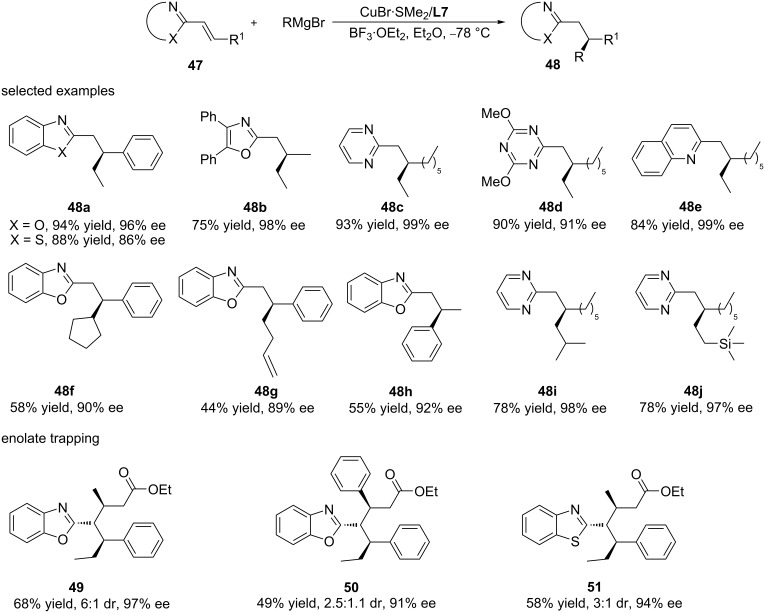
Copper-catalysed ACA of Grignard reagents to alkenyl-substituted aromatic N-heterocycles.

Chiral heterocyclic aromatic compounds are crucial motifs in natural products and bioactive molecules, and in recent years, many strategies have been reported for their highly enantioselective synthesis. However, while catalytic asymmetric C–C bond formations by ACAs of organometallics is a routine procedure for additions to common Michael acceptors, such as enones and enoates, examples of catalytic asymmetric additions to N-heteroaromatic alkenyl compounds are less developed. This deficiency is largely due to the intrinsically low reactivity of alkenyl-substituted heterocycles towards nucleophilic addition compared to common Michael acceptors. A way to lift this barrier was introduced in 2016 by Harutyunyan and co-workers, who developed a general methodology for the direct and facile access to a variety of chiral heterocyclic aromatic compounds by the ACA of Grignard reagents to conjugated N-heteroaromatic alkenyl compounds (Scheme 14) [44]. The key of the presented method was the enhancement of the reactivity of the heteroaromatic alkenyl substrates by Lewis acid activation in combination with readily available and highly reactive Grignard reagents and a copper catalyst bound to a chiral diphosphine ligand. Using this methodology, various chiral heteroaromatic products were obtained with high enantioselectivity (up to 99% ee) and yield (up to 95%). Remarkably, both alkyl and aromatic Grignard reagents provided a high yield and enantioselectivity in this methodology. Furthermore, the same group reported a one-pot conjugate addition to alkenylheteroarenes with subsequent trapping of the resulting azaenolate with reactive Michael acceptors in a follow-up study [45].

While pyridines are among the most important classes of heterocyclic moieties that occur in many bioactive molecules, such as natural products, pharmaceuticals, and agrochemicals, the initial report by the Harutyunyan group did not include alkenylpyridines in the substrate scope. The reason for this was the markedly lower reactivity of alkenylpyridines towards nucleophilic addition as compared to other alkenylheteroarenes.

**Scheme 15:**
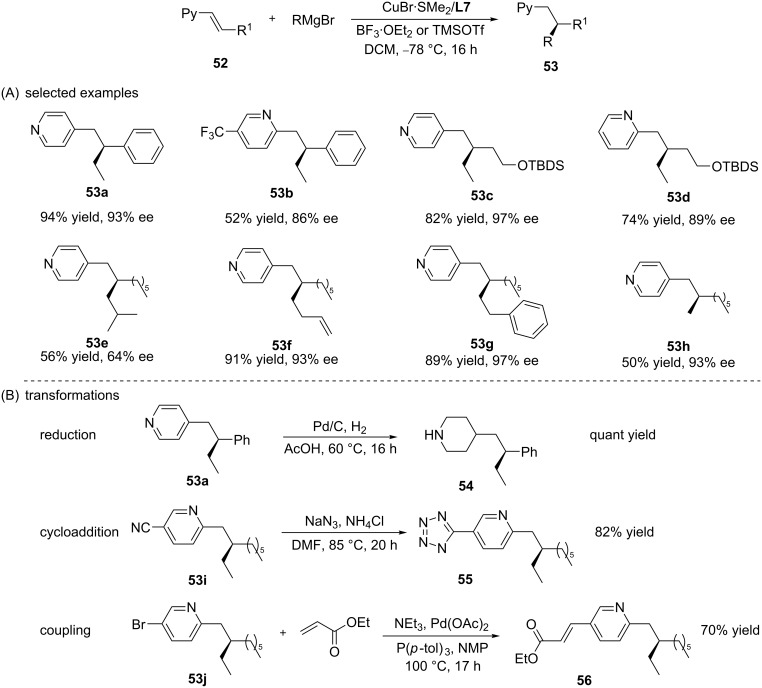
Copper-catalysed ACA of Grignard reagents to β-substituted alkenylpyridines.

For the same reason, the synthesis of chiral pyridine derivatives has always been considered a challenge in organic chemistry research. In an attempt to overcome this reactivity issue, the same authors decided to use the trimethylsilyl-based Lewis acid TMSOTf in order to allow the covalent activation of the alkenylpyridine via pyridinium formation. This strategy turned out successful, and optimisation studies identified reaction conditions that allowed highly enantioselective ACAs of Grignard reagents to alkenylpyridines (Scheme 15) [46]. Using the optimised conditions (Cu/**L7**/TMSOTf), a large variety of pyridine-based chiral compounds was synthesized. Apart from allowing the introduction of different linear, branched, cyclic, and functionalised alkyl chains at the β-position of the alkenylpyridines, the catalytic system also showed a high functional group tolerance, and thus allowing straightforward chemical transformations of the addition products, including, for example, reductions, cycloadditions, and coupling reactions (Scheme 15B).

**Scheme 16:**
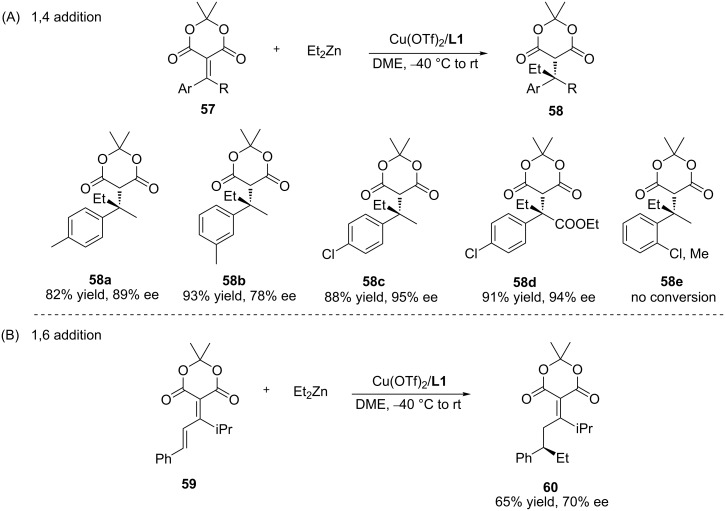
Copper-catalysed ACA of organozinc reagents to alkylidene Meldrum’s acids.

Meldrum’s acid and its derivatives are versatile reagents in organic synthesis that can be transformed into a wide range of compounds. In 2006, the group of Fillion described the highly enantioselective synthesis of all-carbon benzylic quaternary stereocentres via a conjugate addition of dialkylzinc reagents to alkylidene Meldrum’s acids, resulting in the ACA products with high enantiopurity (Scheme 16) [47–52]. Different kinds of Meldrum’s acid derivatives were tolerated in this reaction, and the products could undergo various chemical transformations (Scheme 16A). Later on, this methodology was also demonstrated to enable the 1,6-addition of dialkylzinc reagents to functionalized alkylidene Meldrum’s acids, providing the resulting products **60** with moderate yields (65%) and enantioselectivity (70% ee) (Scheme 16B).

## Conclusion

The aim of this review was to give the reader an overview on the progress made over the past two decades in the field of copper-catalysed C–C bond-forming reactions between heterocyclic acceptors and organometallics. Many excellent methodologies have been reported to date, and the key to the success of these transformations lies in the capability of chiral copper catalysts to activate both the organometallics and heterocyclic acceptors for the reaction. The development of a wide variety of chiral ligands allowed an impressive scope of heterocycles to undergo reactions with organometallics. However, the current state of the field is certainly incomplete, and future developments in substrate and organometallics scope can be expected.
